# Calpastatin-Mediated Inhibition of Calpain Ameliorates Skin Scar Formation after Burn Injury

**DOI:** 10.3390/ijms22115771

**Published:** 2021-05-28

**Authors:** Cheong Hoon Seo, Hui Song Cui, June-Bum Kim

**Affiliations:** 1 Department of Rehabilitation Medicine, Hangang Sacred Heart Hospital, Hallym University College of Medicine, 12 Beodeunaru-ro 7-gil, Yeongdeungpo-gu, Seoul 07247, Korea; mistraldoil@gmail.com; 2 Burn Institute, Hangang Sacred Heart Hospital, Hallym University College of Medicine, 12 Beodeunaru-ro 7-gil, Yeongdeungpo-gu, Seoul 07247, Korea; robbin5217@hanmail.net; 3 Department of Pediatrics, Hangang Sacred Heart Hospital, Hallym University College of Medicine, 12 Beodeunaru-ro 7-gil, Yeongdeungpo-gu, Seoul 07247, Korea

**Keywords:** wound, hypertrophy, complication

## Abstract

Hypertrophic scars, the most common complication of burn injuries, are characterized by excessive deposition of fibroblast-derived extracellular matrix proteins. Calpain, a calcium-dependent protease, is involved in the fibroblast proliferation and extracellular matrix production observed in certain fibrotic diseases. However, its role in the formation of post-burn hypertrophic skin scars remains largely unknown. Here, calpain expression and activity were assessed in skin fibroblasts obtained directly from patients with third-degree burns, who consequently developed post-burn hypertrophic scars. Furthermore, the antifibrotic effect of calpastatin, an endogenous calpain inhibitor, was evaluated in human fibroblasts and a murine burn model. The activity, mRNA levels, and protein levels of calpain were markedly higher in fibroblasts from the burn wounds of patients than in normal cells. Selective calpain inhibition by calpastatin markedly reduced not only the proliferation of burn-wound fibroblasts but also the mRNA and protein expression of calpain, transforming growth factor-beta 1, α-smooth muscle actin, type I and type III collagens, fibronectin, and vimentin in burn-wound fibroblasts. The anti-scarring effects of calpastatin were validated using a murine burn model by molecular, histological, and visual analyses. This study demonstrates the pathological role of calpain and the antifibrotic effect of calpastatin via calpain inhibition in post-burn hypertrophic scar formation.

## 1. Introduction

The formation of post-burn hypertrophic scars is the most common complication of burn injuries that deteriorates the patient’s quality of life based on cosmetic, functional, and psychosocial perspectives [1,2]. Most patients with severe third-degree burns have hypertrophic scars for which effective treatments have not been established [3]. Hypertrophic scars are a side effect of fibroproliferative diseases resulting from the constitutive proliferation of dermal fibroblasts and their excessive production of extracellular matrix (ECM) proteins, including collagen [1,3]. Fibroblasts play pivotal roles in wound healing and serve as major drivers in the fibrotic process of hypertrophic scar formation [4,5].

Calpains are Ca^2+^-dependent proteases involved in various cellular functions, including cell proliferation and differentiation, tissue remodeling, and fibrosis [6,7,8]. Their functionalities are attributed primarily to two major isoforms conventionally known as calpain-1 and calpain-2, which are activated in response to micromolar and millimolar concentrations of Ca^2+^, respectively [9]. The pathological overactivation of calpains has been implicated in certain fibrotic diseases, including cardiac fibrosis and idiopathic pulmonary fibrosis [10,11,12]. Calpain upregulates transforming growth factor-beta 1 (TGF-β1) both at the transcriptional and post-translational levels to induce profibrotic activities, including fibroblast proliferation and ECM synthesis, via Smad2/3 and non-Smad (Akt) signaling. Furthermore, TGF-β1 positively regulates calpain [12,13,14]. This mechanism can induce the pathological accumulation of fibroblasts and ECM, resulting in tissue fibrosis.

To suppress the deleterious effects of calpain, its activity is regulated by calpastatin, a selective endogenous inhibitor [9]. Calpastatin is a highly selective and potent inhibitor of calpains and has been widely used to study the physiological function of calpains [15,16]. Transgenic overexpression of calpastatin was reported to markedly decrease myocardial hypertrophy, fibrosis, and dysfunction [17,18,19]. Calpastatin can affect cellular functions not only via intracellular but also through extracellular routes [20]. For instance, the proliferation and migration of pulmonary arterial smooth muscle cells are reduced by exogenously administering non-cell-permeable calpastatin [21].

Although calpain and calpastatin have been implicated in several fibrotic disorders, their roles in hypertrophic skin scarring after burn injury remain largely unknown. In this study, we examined the early-stage pathogenesis of post-burn hypertrophic scar formation by investigating the expression and activity of calpains in skin fibroblasts of patients, 1–2 weeks after developing third-degree burns. Using in vitro human specimens and in vivo mouse models, we also evaluated whether calpain inhibition by calpastatin could prevent the development of burn skin scars.

## 2. Results

### 2.1. Calpain Activity and Expression Are Increased in Burn-Wound Fibroblasts (BFs) Obtained from Patients

**Figure 1:**
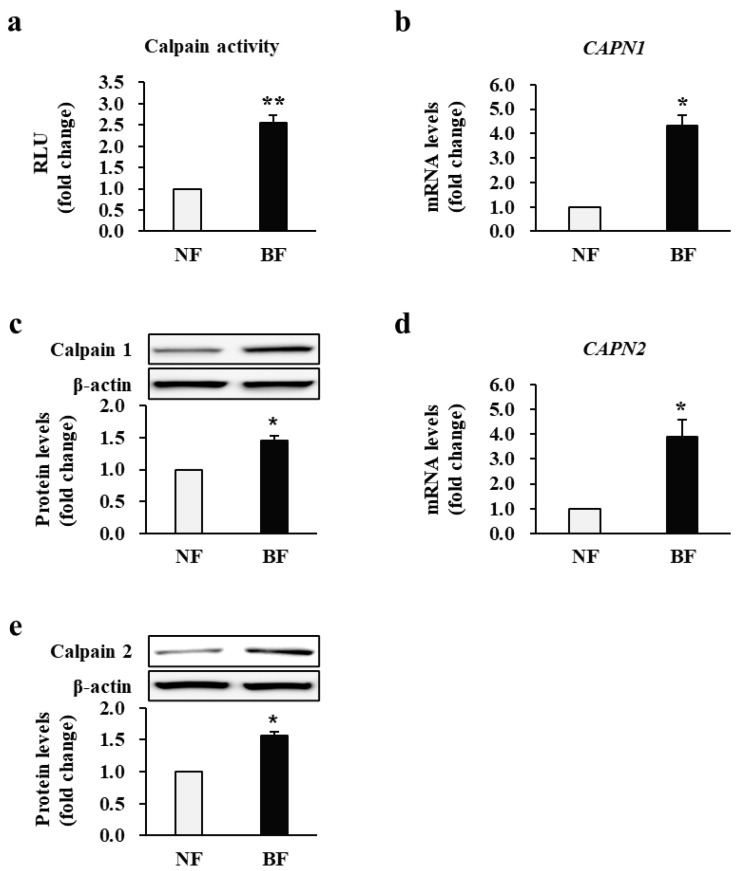
Activity and expression of calpain in fibroblasts obtained from patients with burn injuries. (**a**) Calpain activity in burn-wound fibroblasts (BF) and normal fibroblasts (NF). The mRNA and protein levels of (**b**,**c**) calpain-1 and (**d**,**e**) calpain-2 in NFs and BFs. Calpain activity and all expression levels in BFs were compared with and normalized to those in NFs for each patient. RLU: Relative light units. * p < 0.05 and ** p < 0.01. Data represent mean ± standard error of the mean; *n* = 34 (NF) and *n* = 34 (BF).

As calpains are thought to be responsible for the healing of wounds, we assessed the calpain activity and expression in BFs and normal fibroblasts (NFs) derived from all patient samples. NFs were derived from non-burn skin tissues used for split-thickness skin autografts. Calpain activity was remarkably higher in BFs than in NFs (2.53 ± 0.18-fold increase; *p* < 0.01; NF *n* = 34, BF *n* = 34; Figure 1a). As calpain activity is primarily driven by calpain-1 and calpain-2, their mRNA and protein expression levels were estimated by quantitative reverse transcription-polymerase chain reaction (qRT-PCR) and Western blotting, respectively, as shown in Figure 1b–e. BFs showed a significantly higher mRNA and protein expression of these proteins compared to NFs (calpain-1 mRNA: 4.30 ± 0.41, protein: 1.46 ± 0.07-fold; calpain-2 mRNA: 3.9 ± 0.70, protein: 1.54 ± 0.07-fold increase in BFs vs. NFs; *p* < 0.05; NF *n* = 34, BF *n* = 34; Figure 1b–e). Each experiment was performed in triplicate.

### 2.2. Fibrotic Marker Expression Is Increased at Both Transcriptional and Translational Levels in Patient BFs

**Figure 2:**
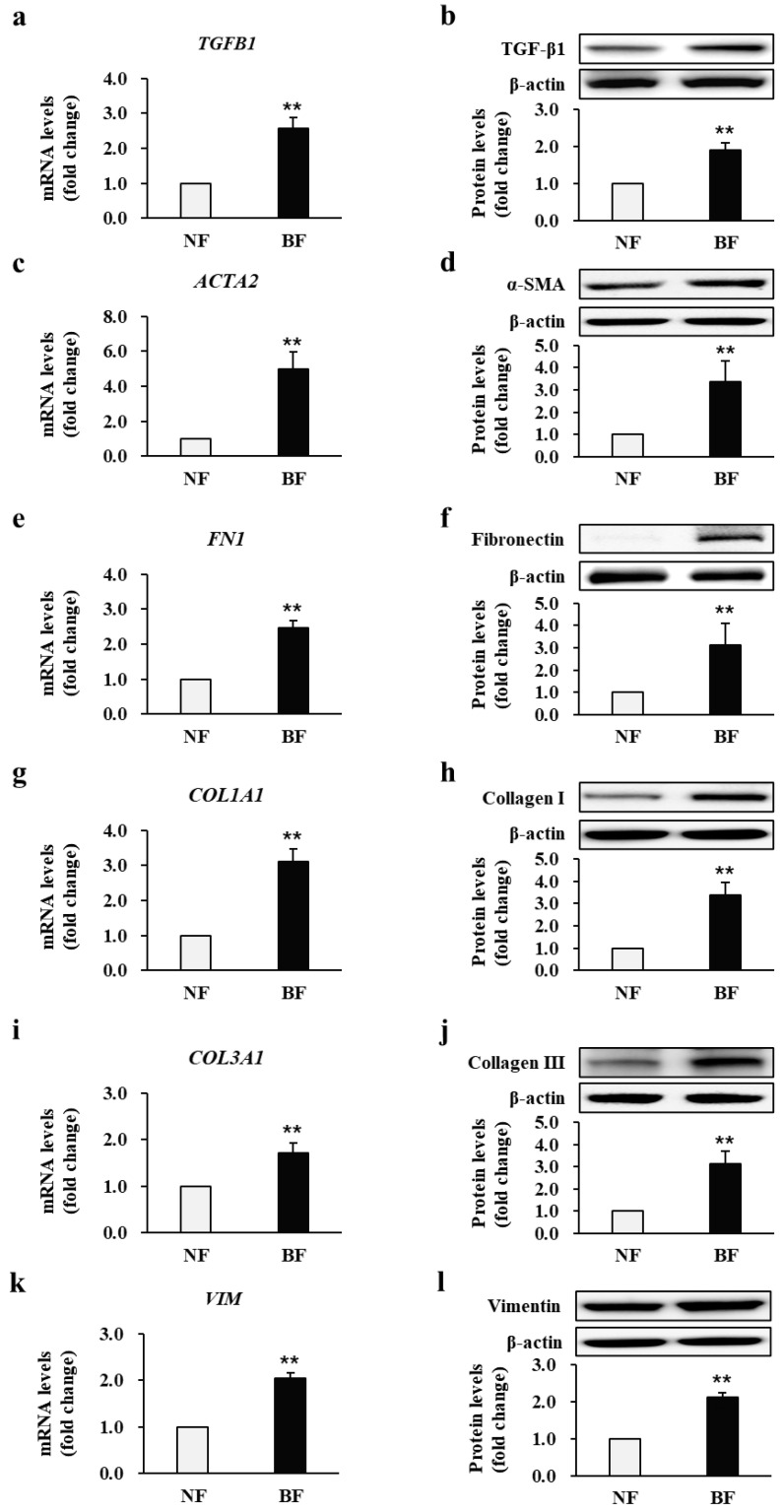
Transcriptional and translational levels of fibrotic markers. The mRNA and protein levels of (**a**,**b**) TGF-β1 (*TGB1*), (**c**,**d**) α-SMA (*ACTA2*), (**e**,**f**) fibronectin (*FN1*), (**g**,**h**) collagen I (*COL1A1*), (**i**,**j**) collagen III (*COL3A1*), and (**k**,**l**) vimentin (*VIM*) in burn-wound fibroblasts (BF) were compared with and normalized to those in normal fibroblast (NF) expression levels for each patient. ** *p* < 0.01. Data represent mean ± standard error of the mean; *n* = 34 (NF) and *n* = 34 (BF).

To investigate the expression profile of fibrotic markers in patients’ fibroblasts following damage inflicted due to third-degree burns, the transcriptional and translational levels of TGF-β1 (*TGB1*), α-smooth muscle actin (α-SMA) (*ACTA2*), fibronectin (*FN1*), collagen I (*COL1A1*), collagen III (*COL3A1*), and vimentin (*VIM*) were examined using qRT-PCR and Western blotting, respectively. Figure 2a,c,e,g,i,k shows that the mRNA expression of TGF-β1, α-SMA, fibronectin, collagen I, collagen III, and vimentin was significantly higher in BFs than that in NFs from all patients who developed post-burn hypertrophic scars (2.56 ± 0.32, 4.98 ± 0.98, 2.46 ± 0.21, 3.11 ± 0.36, 1.72 ± 0.21, and 2.05 ± 0.12-fold increase, respectively; *p* < 0.01; NF *n* = 34, BF *n* = 34). The protein expression levels of TGF-β1, α-SMA, fibronectin, collagen I, collagen III, and vimentin, as shown in Figure 2b–l, were significantly higher in BFs than those in NFs from all patients who developed post-burn hypertrophic scars (1.89 ± 0.21, 3.36 ± 0.95, 3.13 ± 0.98, 3.38 ± 0.58, 3.12 ± 0.57, and 2.12 ± 0.13-fold increase, respectively; *p* < 0.01; NF *n* = 34, BF *n* = 34). Each experiment was performed in triplicate.

### 2.3. Calpastatin Inhibits In Vitro Proliferation of Patient BFs

**Figure 3:**
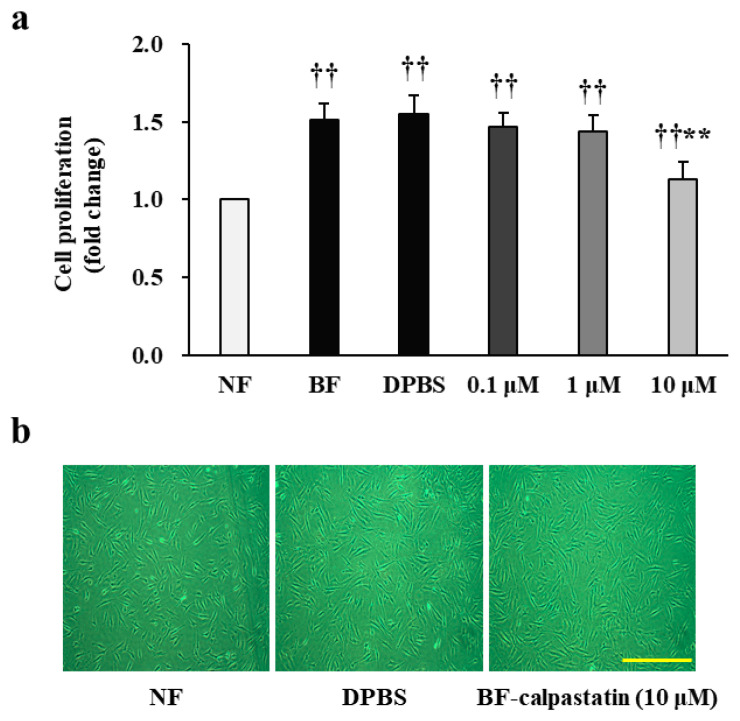
Effect of calpastatin on the proliferation of fibroblasts obtained from patients with burn injuries. (**a**) Proliferation observed in normal fibroblasts (NF), burn-wound fibroblasts (BF), Dulbecco’s phosphate-buffered saline (DPBS)-treated BFs (DPBS), and BFs treated with calpastatin at different concentrations (0.1, 1, and 10 μM) for 48 h. (**b**) Cell images (10×) showing NFs, DPBS-treated BFs, and calpastatin (10 μM)-treated BFs. Scale bar = 50 μm. Proliferation of BFs upon calpastatin treatment was compared with and normalized to that of NFs or DPBS-treated BFs for each patient. ^††^ *p* < 0.01 vs. NFs; ** *p* < 0.01 vs. DPBS-treated BFs. Data are expressed as mean ± standard error of the mean, *n* = 34 (each group).

Then, we examined whether calpastatin, a known calpain inhibitor, can attenuate the proliferation of BFs derived from all patients who developed post-burn hypertrophic scars. First, the proliferation of patient BFs and NFs was assessed after 48 h of culture by performing a colorimetric assay (Figure 3a,b), which revealed a significantly higher increase in BFs (1.51 ± 0.11-fold increase; *p* < 0.01). Subsequently, the effect of calpastatin was estimated by supplying it to primary fibroblasts at various concentrations along with the respective vehicular control and quantifying the differences in proliferation, as shown in Figure 3a,b. BFs exposed to 0.1 or 1 μM calpastatin for 48 h showed no significant change in proliferation as compared to those treated with Dulbecco’s phosphate-buffered saline (DPBS) alone (0.1 and 1 μM: 0.97 ± 0.04-fold and 0.95 ± 0.05-fold decrease, respectively; *p* > 0.05). The treatment with 10 μM calpastatin significantly inhibited the proliferation of BFs compared to that of BFs treated with DPBS alone (0.75 ± 0.06-fold decrease; *p* < 0.01). However, the proliferation of BFs treated with 10 μM calpastatin remained higher than that of NFs (1.13 ± 0.12-fold increase; *p* < 0.01). Each cell group (*n* = 34 per group) was tested in triplicate.

### 2.4. Calpastatin Inhibits Calpain Activity and Expression in Patient BFs

**Figure 4:**
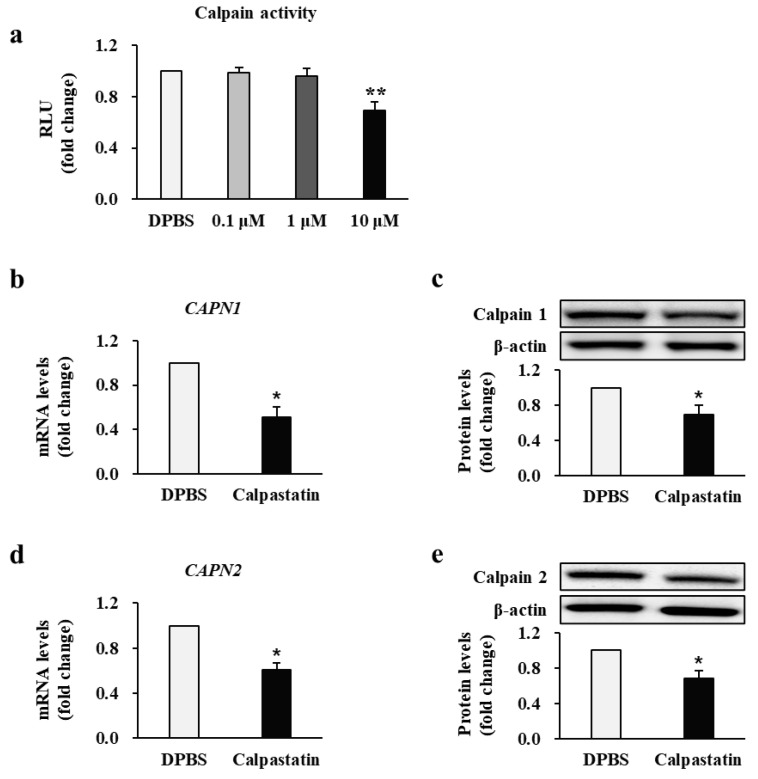
Effect of calpastatin on the activity and expression of calpain in burn-wound fibroblasts (BFs) of patients. (**a**) Calpain activity and mRNA and protein expression levels of (**b**,**c**) calpain-1 and (**d**,**e**) calpain-2 in BFs treated with 10 μM calpastatin for 48 h compared with those in Dulbecco’s phosphate-buffered saline (DPBS)-treated BFs. Activity and expression levels were normalized to those of DPBS-treated BFs for each patient. DPBS: DPBS-treated BFs; Calpastatin: Calpastatin-treated BFs; RLU: Relative light units. * *p* < 0.05 and ** *p* < 0.01. Data are expressed as mean ± standard error of the mean, *n* = 34 (each group).

Next, we examined whether calpastatin directly affects calpain activity and expression in BFs from all patients who developed post-burn hypertrophic scars. Thus, we evaluated calpain activity after treating BFs with calpastatin. As shown in Figure 4a, calpain activity decreased significantly in BFs treated with 10 μM calpastatin compared to DPBS-treated BFs (0.69 ± 0.05-fold decrease; *p* < 0.01). The treatment with 0.1 or 1 μM calpastatin had no significant inhibitory effect (0.1 μM: 0.98 ± 0.04-fold and 1 μM: 0.96 ± 0.06-fold decrease, respectively; *p* > 0.05). The effect of calpastatin on mRNA and protein expression levels of calpain was measured (Figure 4b–e). BFs treated with 10 μM calpastatin for 48 h showed significantly lower mRNA and protein expression levels of calpain-1 and calpain-2 compared to those in DPBS-treated BFs (calpain-1 mRNA: 0.51 ± 0.09, protein: 0.63 ± 0.11-fold; calpain-2 mRNA: 0.61 ± 0.05, protein: 0.67 ± 0.09-fold decrease in calpastatin-treated BFs relative to levels in DPBS-treated BFs; *p* < 0.05). These results suggest that calpastatin negatively regulates calpain expression and activity in BFs. Each cell group (*n* = 34 per group) was tested in triplicate. 

### 2.5. Calpastatin Inhibits Fibrotic Marker Expression in Patient BFs

**Figure 5:**
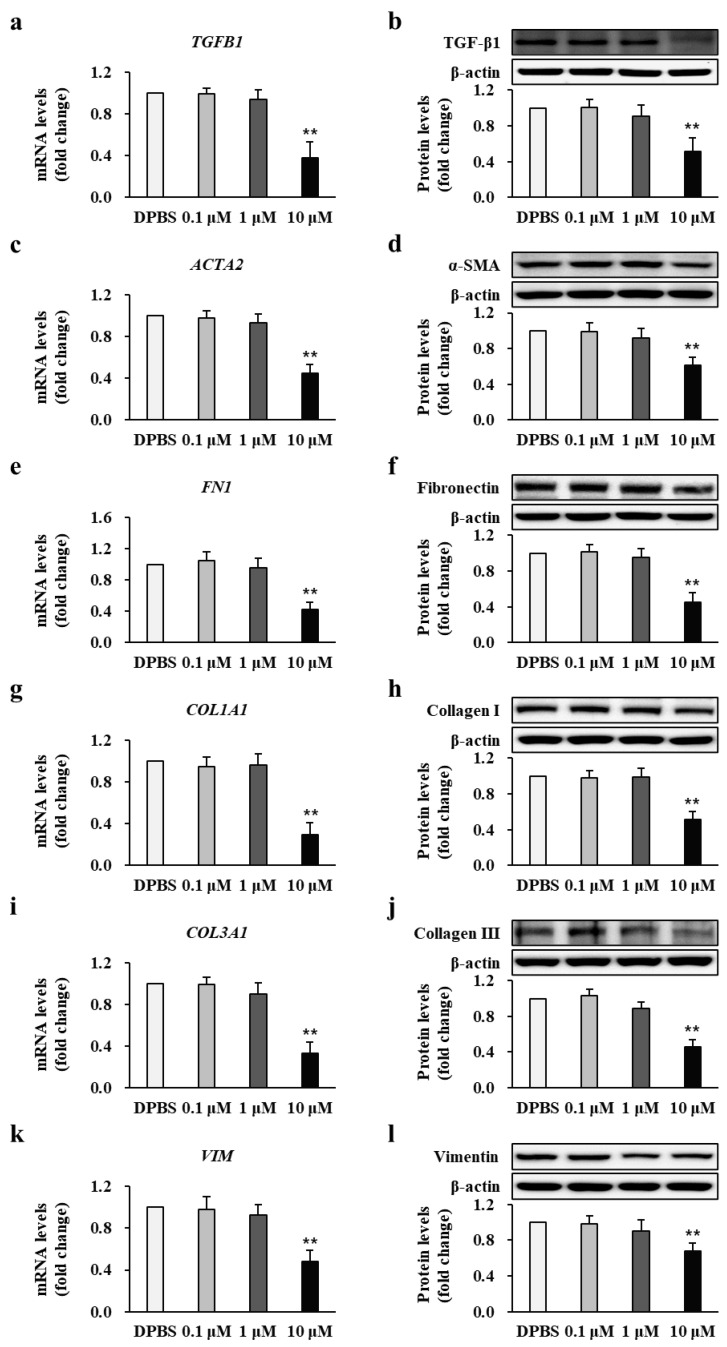
Effect of calpastatin on fibrotic marker expression in burn-wound fibroblasts (BFs) of patients. The mRNA and protein levels of (**a**,**b**) TGF-β1 (*TGB1*), (**c**,**d**) α-SMA (*ACTA2*), (**e**,**f**) fibronectin (*FN1*), (**g**,**h**) collagen I (*COL1A1*), (**i**,**j**) collagen III (*COL3A1*), and (**k**,**l**) vimentin (*VIM*) were significantly decreased in 10 μM calpastatin-treated BFs compared with Dulbecco’s phosphate-buffered saline (DPBS)-treated BFs (DPBS). All expression levels were normalized to those in DPBS-treated BFs for each patient. ** *p* < 0.01. Data represent mean ± standard error of the mean; *n* = 34 (each group).

As calpastatin was demonstratedAs calpastatin was demonstrated to affect calpain activity, we next examined its effect on fibrotic markers by analyzing the mRNA and protein expression of TGF-β1, α-SMA, fibronectin, collagen I, collagen III, and vimentin in BFs treated with calpastatin and its respective vehicular control BFs derived from all patients (*n* = 34) who developed post-burn hypertrophic scars. Patient BFs treated with 10 μM calpastatin for 48 h showed significantly lower mRNA and protein expression levels of TGF-β1 (0.38 ± 0.15 and 0.51 ± 0.16-fold decrease, respectively; *p* < 0.01) (Figure 5a,b), α-SMA (0.45 ± 0.08 and 0.61 ± 0.09-fold decrease, respectively; *p* < 0.01) (Figure 5c,d), fibronectin (0.42 ± 0.10 and 0.45 ± 0.11-fold decrease, respectively; *p* < 0.01) (Figure 5e,f), collagen I (0.29 ± 0.12 and 0.51 ± 0.09-fold decrease, respectively; *p* < 0.01) (Figure 5g,h), collagen III (0.33 ± 0.11 and 0.43 ± 0.08-fold decrease, respectively; *p* < 0.01) (Figure 5i,j), and vimentin (0.44 ± 0.11 and 0.67 ± 0.09-fold decrease, respectively; *p* < 0.01) (Figure 5k,l) compared to those treated with DPBS. Each cell group (*n* = 34 per group) was tested in triplicate. 

### 2.6. Calpastatin Suppresses Fibrotic Marker Expression in Mouse Burn-Wound Tissues

**Figure 6:**
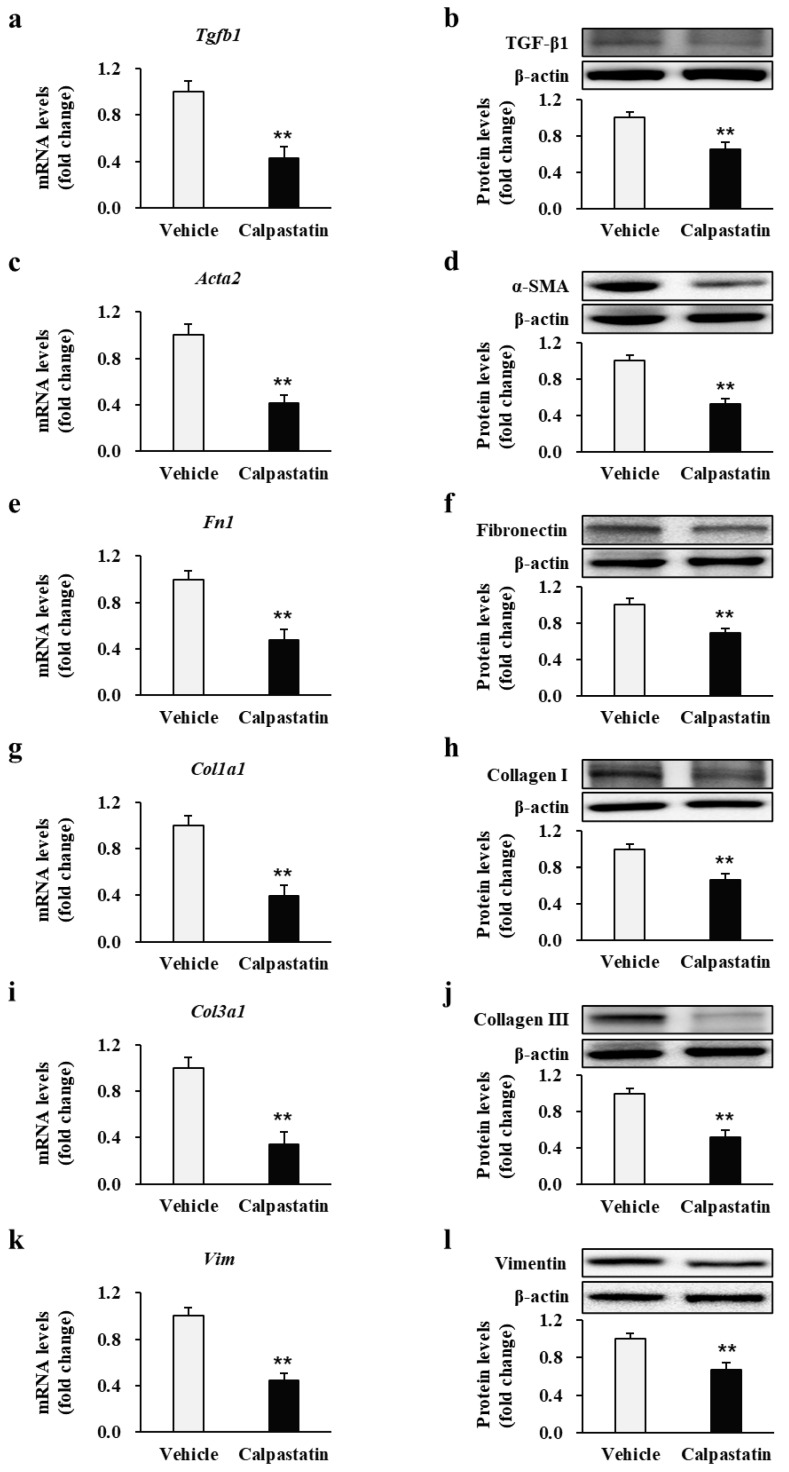
Effect of calpastatin on fibrotic marker expression in burn wounds of mice. The mRNA and protein levels of (**a**,**b**) TGF-β1, (**c**,**d**) α-SMA, (**e**,**f**) fibronectin, (**g**,**h**) collagen I, (**i**,**j**) collagen III, and (**k**,**l**) vimentin in burn-wound tissues of mice administrated calpastatin once daily for 28 d post-burn injury were compared with those in vehicle-treated mice. All expression levels were normalized to those in vehicle-treated mice, in which the mean value for vehicle controls was designated as value 1. ** *p* < 0.01. Data represent mean ± standard error of the mean; *n* = 36 (vehicle) and *n* = 36 (calpastatin).

To further explore the efficacy of the antifibrotic effects of calpastatin, a murine burn model was generated, and the mice were administered calpastatin to determine its effects on fibrotic markers in burn fibroblasts in vivo, as shown in Figure 6. Burn-wound tissues from 36 mice administered calpastatin (1.5 μg/kg/day, intraperitoneally (i.p.) for 28 d had significantly decreased mRNA and protein expression levels of TGF-β1 (0.42 ± 0.09 and 0.65 ± 0.07-fold decrease, respectively; *p* < 0.01) (Figure 6a,b), α-SMA (0.41 ± 0.07 and 0.52 ± 0.06-fold decrease, respectively; *p* < 0.01) (Figure 6c,d), fibronectin (0.47 ± 0.09 and 0.69 ± 0.05-fold decrease, respectively; *p* < 0.01) (Figure 6e,f), collagen I (0.39 ± 0.09 and 0.66 ± 0.07-fold decrease, respectively; *p* < 0.01) (Figure 6g,h), collagen III (0.33 ± 0.11 and 0.52 ± 0.07-fold decrease, respectively; *p* < 0.01) (Figure 6i,j), and vimentin (0.43 ± 0.06 and 0.67 ± 0.07-fold decrease, respectively; *p* < 0.01) (Figure 6k,l) compared with the levels in the vehicle controls (*n* = 36). The number of tissue samples is 36 from each group. Each experiment was performed in triplicate.

### 2.7. Calpastatin Inhibits Post-Burn Scar Formation in Mice

**Figure 7:**
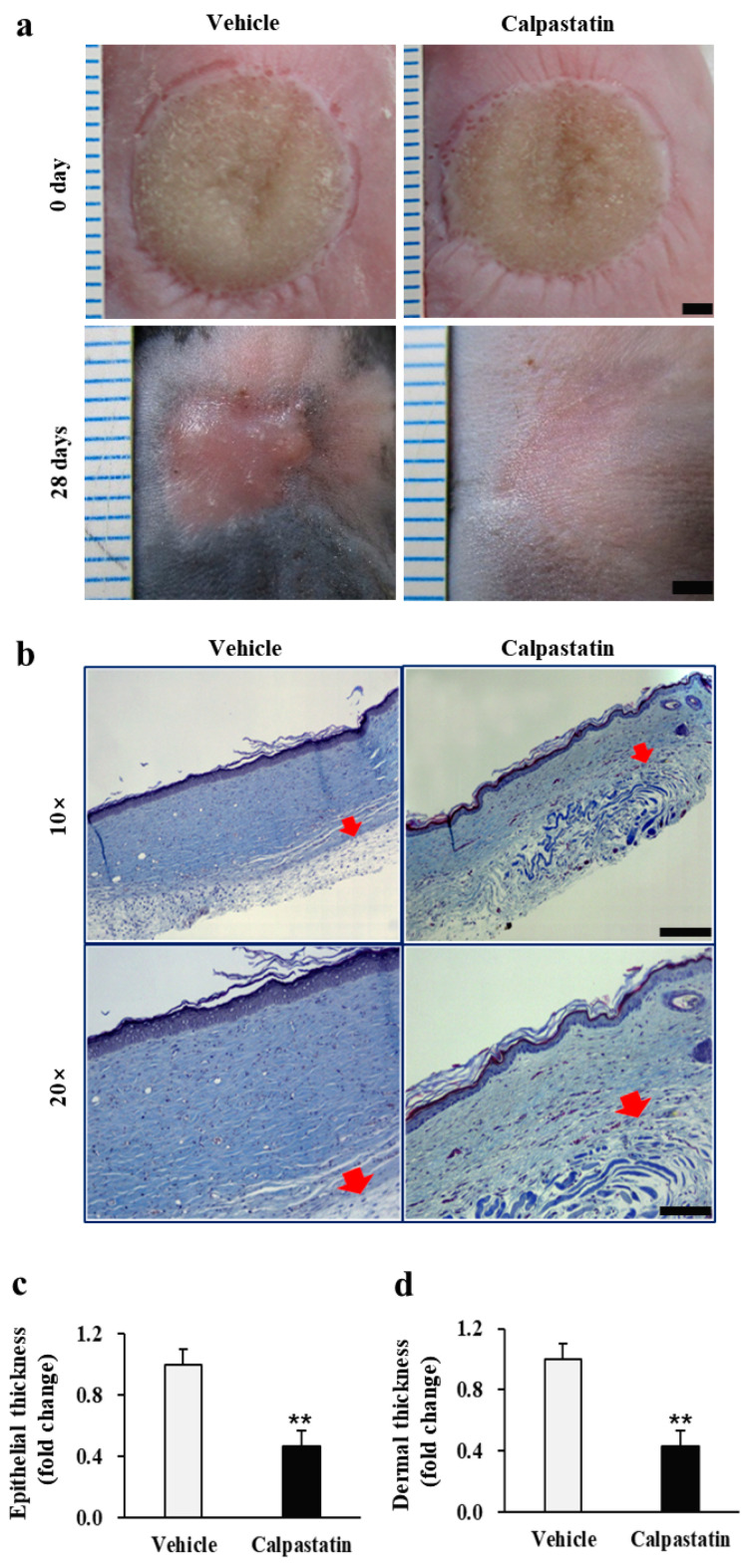
Effect of calpastatin on post-burn scar formation in mice. (**a**) Wound images of vehicle control and calpastatin-treated mice showing less discoloration and irregular thickening in the burn skin wound after the calpastatin treatment. Unit of rulers, 1 mm. Scale bars = 2 mm. (**b**) Histological images of burn skin wounds of calpastatin-treated mice obtained by Masson’s trichrome staining showing decreased epidermal and dermal thicknesses. Scale bar = 100 μm at 10×. The arrows indicate the border between the dermis and subcutaneous tissue. Quantitative analysis of (**c**) epidermal and (**d**) dermal thicknesses of vehicle-treated (vehicle) and calpastatin-treated (calpastatin) burn wounds in mice. Thicknesses were normalized to those of the vehicle-treated mice, in which the mean value of vehicle controls was set as value 1. ** *p* < 0.01. Data represent mean ± standard error of the mean; *n* = 36 (vehicle) and *n* = 36 (calpastatin).

The most important consequence of calpastatin treatment of burn wounds is its potential to limit post-burn scar formation augmented by calpain. Therefore, to verify whether calpastatin plays an inhibitory role in burn scar formation, the burn wounds of mice treated with calpastatin and the respective vehicular control were analyzed. As shown in Figure 7a, the burn wounds of calpastatin-treated mice were visually less erythematous, flatter, and more flexible than those of vehicle-treated mice, which showed erythematous and irregular thickening, and rigidity 28 d after burn induction. Histological findings obtained as a result of Masson’s trichrome staining revealed decreased (almost two times less) epidermal and dermal thicknesses of burn wounds in calpastatin-treated mice on day 28 than those in the vehicle-treated controls (0.45 ± 0.10 and 0.42 ± 0.11-fold, respectively; *p* < 0.01; 108 tissue sections (three sections each from 36 mice per group) in each group) (Figure 7b–d; Supplementary Figure S1). No relative difference was observed between the epidermal thickness and dermal thickness of burn wounds between calpastatin- and vehicle-treated mice.

## 3. Discussion

Approximately 70% of the burn patients, including a majority of those with third-degree burns, develop hypertrophic scars, which are associated with unfavorable cosmetic, functional, and psychological consequences. However, no effective therapy for preventing post-burn hypertrophic scar formation is available [1,2,3]. Wound healing occurs in three distinct but overlapping phases, namely inflammation, proliferation, and remodeling. During the inflammatory step, numerous cytokines and other pro-inflammatory factors are released into the wound site to restore the tissue integrity. The proliferation phase begins several days after injury, with fibroblast infiltration and proliferation, followed by deposition of collagen into the wound area, which is critical for hypertrophic scar formation [3]. To explore the early post-burn stage mechanism of hypertrophic scar formation, we obtained fibroblasts directly from patients within 1–2 weeks of developing third-degree burns; they subsequently developed post-burn hypertrophic scars. Our findings revealed increased cell proliferation and higher expression of hypertrophic markers in human BFs. These results suggest that BFs are activated after developing third-degree burns and are associated with the pathogenesis of post-burn hypertrophic scar formation. We also observed increased activity and expression of calpain in BFs. Calpains are activated in response to increased cytosolic Ca^2+^ levels. Studies have shown that cytosolic Ca^2+^ levels play a pivotal role in wound healing and hypertrophic scar formation. Additionally, Ca^2+^ channel blockers inhibit platelet-derived growth factor-dependent collagen deposition in fibroblasts [22,23]. Whether calpains and platelet-derived growth factor interact in collagen deposition during the wound healing process requires further analysis.

Calpains participate in various physiological processes, including apoptosis, cell mobility, cell cycle progression, and long-term potentiation of neurons [6,7,8]. Calpains are also involved in the pathogenesis of several remodeling-related fibrosis diseases. In vitro studies have shown that calpains markedly increase collagen I synthesis in human lung fibroblasts when their activity is enhanced by bleomycin, and in vivo collagen levels increase in the lung tissues of bleomycin-challenged mice [10,11]. Moreover, calpains play important roles in the development of myocardial interstitial fibrosis by activating TGF-β, which is also important in the pathogenic mechanism of post-burn hypertrophic scar formation [3,12]. Based on these results, we investigated the role of calpain and antifibrotic potential of calpastatin in post-burn hypertrophic scar formation. Our results demonstrate that calpain activity and expression were increased in BFs, and targeted calpain inhibition by calpastatin remarkably reduced cell proliferation and the expression of hypertrophic markers, including collagens, in BFs. The antifibrotic effect of calpastatin was confirmed in murine burn models through molecular, histological, and visual analyses. Increasing evidence has demonstrated that crosstalk occurs between calpain and TGF-β in multiple organs [11,12]. While calpain positively regulates TGF-β1, TGF-β1 can also induce calpain activation. Moreover, inhibition of calpain negatively regulates TGF-β1-mediated Smad2/3 and non-Smad (Akt) signaling pathways [11,12,13,14,24]. We recently reported increased levels of TGF-β1 in fibroblasts obtained from post-burn hypertrophic scars [25]. In the present study, we also observed increased levels of TGF-β1 in BFs obtained from patients early after severe burns that consequently resulted in post-burn hypertrophic scars. Additionally, the calpastatin treatment reduced the expression level of TGF-β1 in BFs from patients and in burn wounds from murine models. Therefore, calpastatin is believed to exert an antifibrotic effect via inhibition of TGF-β1 signaling.

The antifibrotic potential of calpastatin requires further analysis to explore its therapeutic potential in human post-burn hypertrophic scar formation since most existing treatments are used after hypertrophic scar formation. Thus, patients have frequent relapses with limited therapeutic benefits. Wound healing within 3 weeks of a burn is thought to be an important predictor of hypertrophic scarring [26]. Therefore, based on the calpain-related mechanism of scar formation found in this study, early post-burn intervention with calpastatin may prevent hypertrophic scar development itself. We found that the calpastatin treatment did not reduce the proliferation of BFs below the levels observed for NFs. This indicates that calpastatin can suppress burn scar formation without reducing wound healing capacity below that exhibited by normal cells, which has been demonstrated in experiments using murine burn models.

In summary, we identified an increase in the activity and expression of calpains in skin fibroblasts following burn injury and the antifibrotic effect of calpastatin in post-burn scar formation, both in vitro and in vivo, using burn fibroblasts and murine burn models, respectively. These findings indicate that calpain inhibition by calpastatin may be useful for preventing hypertrophic scar formation following burn injuries.

## 4. Materials and Methods

### 4.1. Human Specimens

**Figure 8:**
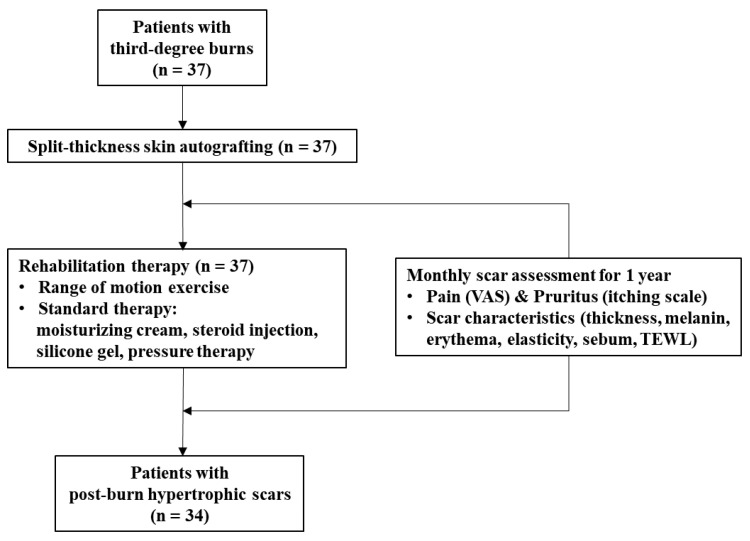
Diagram for patient enrollment and follow-up. VAS: Visual analog scale; TEWL: Transepidermal water loss.

Between 10 January 2018 and 28 March 2019, a total of 37 burn skin tissues and paired normal tissues were collected from each patient receiving split-thickness skin autografting between 1–2 weeks after third-degree burns, at the Burn Institute of Hangang Sacred Heart Hospital, Hallym University, Seoul, Korea (Figure 8). To reduce potential confounding factors, each patient served as their own control by providing some of the normal skin tissue used for autograft from an undamaged part of their body. A sample size was determined based on the number of patients who were available for recruitment during the study period covered by the funding body. The included patients presented with third-degree burns over at least 20% of the total body surface area, with an age range of 8–61 years. To reduce confounding variables influencing wound healing, exclusion criteria were as follows: History of a skin allergy, malignancy, thyroid disease, diabetes, pregnancy or lactation, drug or alcohol abuse, chemotherapy, hormonal therapy, or other prescription drug use within 6 months before burn injury. The post-burn hypertrophic scar development in the patients was assessed individually by two burn surgeons every month for 1 year, and the experimental data obtained after burn injuries in whom hypertrophic scars did not subsequently develop were finally excluded from the study. Of the 37 patients who participated in the present study, post-burn hypertrophic scar formation occurred in 34 patients. 

Table 1 shows the demographic and burn characteristics of the patients in whom post-burn hypertrophic scars were developed. A written informed consent was obtained from all patients donating tissues, and this work was conducted in agreement with the guidelines of the Institutional Review Board of Hangang Sacred Heart Hospital (HG2018-018).

### 4.2. Animals Used

DBA/2NHsd inbred mice weighing 20–25 g and aged 6 weeks were purchased commercially (Koatech Laboratory Animals, Inc., Pyeongtaek, Gyeonggi-do, Korea). The mice were housed in polycarbonate cages and were provided *ad libitum* access to standard laboratory chow and filtered water. The room temperature and relative humidity were 25 ± 2 °C and 55 ± 5%, respectively, and 12-h light/12-h dark cycles were maintained. All animal experiments were performed in the animal laboratory of Hangang Sacred Heart Hospital, Hallym University, in agreement with the Guide for Care and Use of Laboratory Animals of the National Institutes of Health. This study was approved by the Animal Research Ethics Board of Hallym University (HMC2018-2-0222-2).

### 4.3. Fibroblast Isolation and Culture

The isolation and culture of the burn-wound dermal fibroblasts from patients were conducted as previously reported [25]. Skin tissue samples were washed thrice with 70% ethanol and DPBS (Biowest, Riverside, MO, USA), respectively. Thereafter, they were immersed in cold DPBS supplemented with a 1% antibiotic–antimycotic agent consisting of streptomycin, penicillin, and amphotericin B (Gibco, Grand Island, NY, USA). Fine tweezers and a scalpel were used to remove the subcutaneous fat and loose connective tissues. The tissues were cut into small pieces about 1–2 mm wide, transferred to 50 mL conical tubes with a 10 mL Dispase II solution (1 U/mL) (Gibco), and incubated overnight at 4 °C. On the following day, the dermis and epidermis were pulled/peeled using a pair of sterile forceps. The separated dermis was digested using a collagenase type IV solution (500 U/mL) (Gibco) at 37 °C for 30 min. The samples were then immersed in 15 mL Dulbecco’s modified Eagle’s medium (DMEM; Biowest) containing 10% fetal bovine serum (FBS; Biowest) to inactivate the collagenase, filtered using a 100 μm cell strainer (Thermo Fisher Scientific, Waltham, MA, USA), and centrifuged at 300× *g* for 5 min. The pellet was resuspended in DMEM containing 10% FBS and cultured at 37 °C and 5% CO_2_. The expanded fibroblasts were used at passages 1–2 for all experiments.

### 4.4. Cell Proliferation and Calpastatin Treatment

The proliferation of NFs, BFs, and calpastatin-treated BFs derived from patient samples was investigated using the CellTiter 96^®^ AQueous One Solution Cell Proliferation Assay kit (Promega, Madison, WI, USA). NFs were obtained from non-burn skin, which was used for the split-thickness skin autografts. Cells were seeded into 96-well tissue culture plates (2 × 10^4^ cells/well) containing DMEM supplemented with 10% FBS and 1% antibiotic–antimycotic agent. On the next day, the culture medium was replaced with DMEM containing 10% FBS, and BFs were treated for 48 h with 0.1, 1 or 10 μM acetyl-calpastatin (Tocris Bioscience, Bristol, UK) dissolved in DPBS. The fresh culture medium (100 μL DMEM) with 20 μL of CellTiter 96^®^ AQueous One Solution Reagent was added to the cells, followed by incubation at 37 °C for 2 h. Thereafter, the absorbance was measured at 490 nm using a 96-well plate reader (BioTek, Winooski, VT, USA). Cell proliferation was calculated as follows: Proliferation (%) = (sample absorbance − background absorbance)/(control sample absorbance − background absorbance) × 100. After 48 h of calpastatin treatment, cell images were acquired using a light microscope (IX 70, Olympus, Tokyo, Japan). Proliferation of BFs with the calpastatin treatment was compared with and normalized to that of NFs or DPBS-treated BFs for each patient. The experiment was performed in triplicate.

### 4.5. Determination of Calpain Activity

Cells were grown in 100-mm dish plates, rinsed twice with DPBS, and harvested using the cell detachment solution Accutase^TM^ (Invitrogen, Carlsbad, CA, USA). The cells were counted and then lysed with a radioimmunoprecipitation assay (RIPA) buffer (ethylenediaminetetraacetic acid-free) (30 μL/L × 10^5^ cells), followed by centrifugation for 30 min (13,000× *g*, 4 °C). Calpain activity was measured in the supernatants using the Calpain-Glo™ protease assay (Promega) according to the manufacturer’s protocol. Luminescence was measured as relative light units on a Multimode Detector (DTX880, Beckman Coulter, Brea, CA, USA). Each cell group was tested in triplicate. The background was subtracted, and the results were expressed as fold-change relative to that of NFs or DPBS-treated BFs for each patient. 

### 4.6. Murine Burn Models and Calpastatin Treatment

Mice were anesthetized and maintained under 100% oxygen and 2.5% isoflurane (Hana Pharm Co., Seoul, Korea). The dorsum of each mouse was shaved and sterilized with electric clippers and 70% alcohol, respectively. A deep third-degree burn wound 10 mm in diameter was created by laser irradiation at a total energy of 700 ± 10 J (Sellas Evo^TM^, Seoul, Korea). The third-degree burn was confirmed histologically. Immediately after inducing burns, saline (vehicle control) or acetyl-calpastatin (1.5 μg/kg) [27] dissolved in saline was administered i.p. to each mouse group (test: *n* = 36; control: *n* = 36) once daily for 28 d. Burn wounds were dressed daily with Vaseline gauzes sterilized by autoclaving. Burned skin tissue was harvested from mice 28 d after burn injury.

### 4.7. The qRT-PCR Analysis

Total RNA was obtained from fibroblasts and burn-wound tissues by homogenizing the samples in RNAzol (Cancer Rop Co., Seoul, Korea) with a gentleMACS™ Dissociator (Miltenyi Biotec, Bergisch-Gladbach, Germany), followed by extraction using a ReliaPrep^TM^ RNA Miniprep System (Promega) according to the manufacturer’s instructions. RNA concentration was measured on a NanoDrop spectrophotometer (BioTek) and 2 µg RNA was converted to cDNA using a PrimeScript™ RT master mix (Takara, Shiga, Japan). The qRT-PCR was conducted on a LightCycler^®^ 96 (Roche, Basel, Switzerland) using 50 ng cDNA and 0.5 µM primers (Table 2). The reaction conditions were as follows: Initial denaturation at 95 °C for 10 min, 40-cycle amplification at 95 °C for 10 s and 60 °C for 30 s, and melt curve analysis at 95 °C for 5 s, 65 °C for 60 s, and 95 °C for 1 s. The mRNA expression of each gene was normalized to the expression of glyceraldehyde 3-phosphate dehydrogenase (GAPDH) via the 2^−ΔΔCt^ method [28]. The mRNA expression levels in BFs with/without the calpastatin treatment were compared with and normalized to those in NFs or DPBS-treated BFs for each patient. In murine burn models, the mRNA expression levels in all burn-wound tissues (*n* = 36) were compared with and normalized to those in all vehicle controls (*n* = 36), in which the mean for vehicles was set as 1. Each sample was assayed in triplicate. 

### 4.8. Western Blot Analysis

Total protein was acquired from fibroblasts and burn-wounds by homogenizing the samples using a gentleMACS™ Dissociator (Miltenyi Biotec) in a RIPA buffer containing protease and phosphatase inhibitors (Sigma-Aldrich, St. Louis, MO, USA). Western blotting was conducted as previously described [29]. Samples were incubated for 30 min at 4 °C with constant agitation, and then centrifuged for 30 min (13,000× *g*, 4 °C). The protein concentrations of the supernatants were measured with a Quick Start^TM^ Bradford protein assay kit (Bio-Rad, Hercules, CA, USA). Lysates were denatured with a 5× reducing sample buffer (Biosesang, Seongnam, Gyeonggi-do, Korea) by heating for 3 min at 95 °C. The samples were separated by gel electrophoresis, electrotransferred onto polyvinylidene difluoride membranes (EMD Millipore, Billerica, MA, USA), and blocked with 5% (*w*/*v*) skim milk in Tris-buffered saline containing 0.1% Tween-20 for 1 h at 25 °C. The following primary antibodies were used: Polyclonal goat anti-calpain-1 antibody (1:500, cat. no. sc-7531; Santa Cruz Biotechnology, Dallas, TX, USA), monoclonal mouse anti-calpain-2 antibody (1:2000, cat. no. NBP2-46063; Novus Biologicals, Littleton, CO, USA), polyclonal rabbit anti-TGFβ-1 antibody (1:200, cat. no. sc-146, Santa Cruz Biotechnology), polyclonal mouse anti-α-SMA antibody (1:500, cat. no. ab1817; Abcam, Cambridge, UK), monoclonal rabbit anti-fibronectin antibody (1:2000, cat. no. ab6328; Abcam), polyclonal rabbit anti-collagen I antibody (1:1000, cat. no. ab34710; Abcam), monoclonal rabbit anti-collagen III antibody (1:2000, cat. no. ab7778; Abcam), polyclonal mouse anti-vimentin antibody (1:3000, cat. no. ab92547; Abcam), polyclonal rabbit anti-β-actin antibody (1:2000, cat. no. 4967; Cell Signaling Technology, Danvers, MA, USA), and monoclonal mouse anti-β-actin antibody (1:1000, cat. no. SC1616; Santa Cruz Biotechnology). Secondary antibodies included horseradish peroxidase-conjugated goat anti-rabbit IgG antibody (1:1000, cat. no. AP307P; EMD Millipore) and horseradish peroxidase-conjugated goat anti-mouse IgG antibody (1:1000, cat. no. AP308P; EMD Millipore). Images were obtained with a chemiluminescence imaging system (WSE-6100; ATTO, Tokyo, Japan), and the optical density of the bands was measured by the CS Analyzer 4 software (ATTO). Protein expression was normalized to that of β-actin. Protein levels in BFs with/without the calpastatin treatment were compared with and normalized to those in NFs or DPBS-treated BFs for each patient. In murine burn models, protein levels in all burn-wound tissues (*n* = 36) were compared with and normalized to those in all vehicle controls (*n* = 36), in which the mean for vehicles was designated as 1. Each sample was assayed in triplicate.

### 4.9. Histological Evaluation

Mice were sacrificed by inhalation of CO_2_ gas under anesthesia at 28 d after burn injury. Burn-wound tissues, including the panniculus carnosus muscle layer, were cautiously excised, immediately fixed in 4% neutral-buffered formalin, and incubated overnight at 25 °C. Subsequently, the tissues were serially dehydrated in 50, 70, 80, 90, 95, and 100% ethanol, cleared with benzene, and embedded in paraffin blocks. Paraffinized tissue blocks were cut into 5-μm-thick sections and mounted on silane-coated slides (Muto Pure Chemicals, Tokyo, Japan). Three sections were randomly selected from each sample. The sections were dewaxed, rehydrated, and stained with Masson’s trichrome (Abcam) according to the manufacturer’s instructions. The sections were dehydrated, mounted, and covered with coverslips. Images of the sections were acquired with a light microscope (Leica Microsystems GmbH, Wetzlar, Germany) at 10× and 20× magnifications. Epidermal and dermal thicknesses were measured using ImageJ 1.53a (National Institutes of Health, Bethesda, MD, USA; https://imagej.nih.gov/ij/download.html) by measuring the highest and lowest width of the epidermis and dermis in each section and then calculating the average value. Epidermal thickness and dermal thickness of burn wounds of calpastatin-treated mice were compared and normalized with those of vehicle-treated mice, in which the mean for vehicles was designated as value 1. The same numbers of tissue sections of vehicle- and calpastatin-treated mice were used to measure the epidermal thickness and dermal thickness (108 sections (three sections each from 36 mice in each group)).

### 4.10. Statistical Analyses

SPSS Statistics version 24.0 (SPSS, Inc., Chicago, IL, USA) was employed for statistical analyses. Results are represented as mean ± standard error of the mean. The sample number (n) indicates the number of independent biological samples in each experiment. Each sample was assayed in triplicate. Comparisons were performed using the Student’s *t*-test and one-way analysis of variance followed by Tukey’s post hoc multiple comparison test, with *p* < 0.05 or *p* < 0.01 indicating statistical significance. Experimental mice were randomly assigned to be administered with either vehicle or calpastatin using a computerized program Stata version 9.0 (StataCorp LLC, College Station, TX, USA).

## Figures and Tables

**Table 1:** Demographic characteristics of burn patients and the nature of the burns.

Patients (*n* = 34)	Cause	Age (Years)	Gender	% TBSA Burned	Location of Specimens (Burn/Non-Burn)
1	Scald	53	Male	23	Leg/leg
2	Scald	28	Male	27	Thigh/thigh
3	Flame	31	Male	28	Leg/leg
4	Flame	25	Male	22.5	Hand/hand
5	Flame	21	Male	23.5	Abdomen/abdomen
6	Contact	43	Male	27	Arm/arm
7	Contact	25	Male	23	Hand/hand
8	Electrical	36	Male	23	Hand/hand
9	Scald	48	Male	22.5	Face/scalp
10	Flame	8	Male	27	Shoulder/shoulder
11	Contact	15	Male	28	Hand/hand
12	Contact	24	Male	27	Leg/leg
13	Flame	38	Male	23.5	Back/back
14	Flame	56	Male	27	Abdomen/abdomen
15	Contact	31	Male	27	Chest/abdomen
16	Flame	42	Male	22.5	Thigh/thigh
17	Contact	18	Male	23	Hand/hand
18	Flame	49	Male	23	Leg/leg
19	Flame	26	Male	22.5	Thigh/thigh
20	Flame	41	Male	27	Face/scalp
21	Flame	26	Male	23	Abdomen/abdomen
22	Scald	24	Female	28	Leg/leg
23	Contact	33	Female	22.5	Thigh/thigh
24	Flame	61	Female	27	Face/scalp
25	Flame	25	Female	28	Abdomen/back
26	Flame	34	Female	23.5	Breast/abdomen
27	Flame	59	Female	23	Face/scalp
28	Flame	37	Female	22.5	Abdomen/thigh
29	Contact	12	Female	27	Leg/leg
30	Flame	35	Female	23	Arm/arm
31	Scald	57	Female	23	Thigh/thigh
32	Flame	19	Female	22.5	Breast/abdomen
33	Flame	43	Female	27	Leg/leg
34	Scald	31	Female	28	Thigh/thigh

TBSA: Total body surface area.

**Table 2:** The qRT-PCR primer sequences.

Gene	Forward (5′ → 3′)	Reverse (5′ → 3′)	Efficiency (%)
*TGFB1* (h)	GTTAAAAGTGGAGCAGCACG	GAGGTATCGCCAGGAATTGT	99.5
*ACTA2* (h)	CCGACCGAATGCAGAAGGA	ACAGAGTATTTGCGCTCCGAA	101.3
*FN1* (h)	CCAGTCCACAGCTATTCCTG	ACAACCACGGATGAGCTG	100.2
*COL1A1* (h)	ATGTTCAGCTTTGTGGACCTC	CTGTACGCAGGTGATTGGTG	99.8
*COL3A1* (h)	CACTGGGGAATGGAGCAAAAC	ATCAGGACCACCAATGTCATAGG	103.1
*VIM* (h)	AACCGACACTCCTACAAGAT	CAGAAACAAGTTGGTTGGATAC	99.9
*CAPN1* (h)	CCTCCCTCACTCTCAACGAC	CTCCAGAACTCGTTGCCTTC	100.3
*CAPN2* (h)	CTGTAGCTAACTGGCCCATCC	TGGAAATTCGTTCCTTTGCG	101.5
*CAST* (h)	AAAGCGAAGGATTCAGCAAA	TCAAAAGTCACCATCCACCA	100.2
*GAPDH* (h)	CATGAGAAGTATGACAACAGCCT	AGTCCTTCCACGATACCAAAGT	101.2
*Tgfb1* (m)	CACTCCCGTGGCTTCTAGTG	GTCTTGCAGGTGGAGAGTCC	99.3
*Acta2* (m)	CAGATGTGGATCAGCAAACAGGA	GACTTAGAAGCATTTGCGGTGGA	99.8
*Fn1* (m)	ATCATAGTGGAGGCACTGCAGAA	GGTCAAAGCATGAGTCATCTGTAGG	102.1
*Col1a1* (m)	GACATGTTCAGCTTTGTGGACCTC	GGGACCCTTAGGCCATTGTGTA	100.5
*Col3a1* (m)	GCACAGCAGTCCAACGTAGA	TCTCCAAATGGGATCTCTGG	101.8
*Vim* (m)	AAAGCGTGGCTGCCAAGAA	ACCTGTCTCCGGTACTCGTTTGA	103.4
*Gapdh* (m)	CATGGCCTTCCGTGTTCCTA	TGTCATCATACTTGGCAGGTTTCT	103.2

H: Human; m: Mouse.

## Data Availability

The datasets generated and/or analyzed during the current study are available from the corresponding author upon request.

## Supplementary Materials

The following are available online at https://www.mdpi.com/article/10.3390/ijms22115771/s1.
