# Supplementary material for: Calpastatin-Mediated Inhibition of Calpain Ameliorates Skin Scar Formation after Burn Injury
Source: Int J Mol Sci. 2021 May 28;22(11):5771. doi: 10.3390/ijms22115771 (PMC8199077; doi:10.3390/ijms22115771)
Supplement: Supplementary file 1 [file ijms-22-05771-s001.zip › ijms-1235678-supplementary.pdf]

SUPPLEMENTARY MATERIALS

Collagen quantification in burn-wound skin tissues stained with Masson's trichrome

Collagen in burn-wound skin tissues stained with Masson's trichrome (Abcam, Cambridge, UK) was quantified using ImageJ software as previously described [1]. Briefly, images of tissue sections were edited by Adobe Photoshop CS6 software (Adobe Systems, Inc., San Jose, CA, USA) to exclude connective tissues. The new images obtained were analyzed with ImageJ 2.1 (FiJi software, Johannes Schindelin, Albert Cardona, Mark Longair, Benjamin Schmid, and others, https://imagej.net/Fiji/Downloads). Images were processed using the "color deconvolution" in the "Image" > "Color" menu to deconvolute the images into pink, blue, and green components; the blue component was identified as collagen. After setting the threshold, go to "Analyze" and select "Measure". The collagen content in burn-wound tissues of calpastatin-treated mice was compared with and normalized to that in vehicle controls, in which the mean value for vehicle controls was designates as 1. The same number of tissue sections of vehicle- and calpastatin-treated mice was used to quantify the collagen content [108 sections (three sections each from 36 mice in each group)]. For comparisons, Student's *t*-test was used with $P < 0.01$ indicating statistical significance.

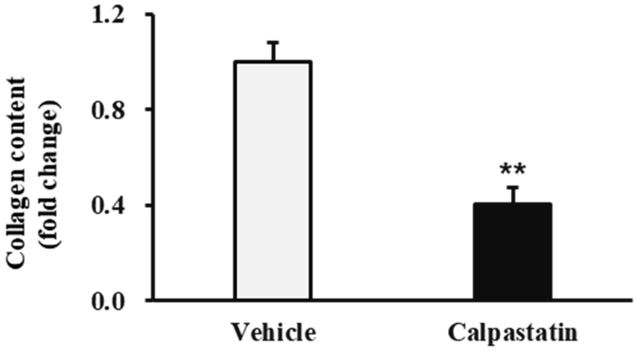

Figure S1. Collagen content in burn-wound skin tissues stained with Masson's trichrome

Collagen quantification in burn-wound skin tissues stained with Masson′s trichrome was determined using ImageJ software. Burn wounds from calpastatin-treated mice had a lower collagen content compared to the vehicle group. Collagen content was normalized to that of the vehicle-treated mice, in which the mean value of vehicle controls was set as value 1. Vehicle, burn wounds from vehicle-treated mice; Calpastatin, burn wounds from calpastatin-treated mice. $**p < 0.01$. Data represent mean ± standard error of the mean; n = 108 (vehicle) and n = 108 (calpastatin). Three sections each from 36 mice in each group.

Reference

[1] Crowe, A.R.; Yue, W. Semi-quantitative Determination of Protein Expression Using Immunohistochemistry Staining and Analysis: An Integrated Protocol. *Bio-protocol.* **2019**, 9, e3465.
